# Antibiogram, Prevalence of OXA Carbapenemase Encoding Genes, and RAPD-Genotyping of Multidrug-Resistant *Acinetobacter baumannii* Incriminated in Hidden Community-Acquired Infections

**DOI:** 10.3390/antibiotics9090603

**Published:** 2020-09-15

**Authors:** Waleed El-Kazzaz, Lobna Metwally, Reham Yahia, Najwa Al-Harbi, Ayat El-Taher, Helal F. Hetta

**Affiliations:** 1 Molecular Microbiology Lab., Botany Department, Faculty of Science, Suez Canal University, Ismailia 41522, Egypt; 2 Microbiology and Immunology Department, Faculty of Medicine, Suez Canal University, Ismailia 41522, Egypt; lobna.metwally@gmail.com; 3 Department of Microbiology, College of Medicine, Taif University, Al-Taif 11099, Saudi Arabia; 4 Basic Sciences Department, College of Science and Health Professions, King Saud Bin Abdul Aziz University for Health Sciences, Riyadh 11481, Saudi Arabia; yahyar@ksau-hs.edu.sa; 5 Molecular Microbiology Lab., King Abdul Aziz University, Riyadh 11481, Saudi Arabia; nmaalharbi@kau.edu.sa; 6 Botany Department, Faculty of Science, Suez Canal University, Ismailia 41522, Egypt; dr_ayateltaher@yahoo.com; 7 Department of Medical Microbiology and Immunology, Faculty of Medicine, Assiut University, Assiut 71515, Egypt; helalhetta@aun.edu.eg; 8 Department of Internal Medicine, University of Cincinnati College of Medicine, Cincinnati, OH 452221, USA

**Keywords:** *Acinetobacter baumannii*, antibiogram, MDR, carbapenemase encoding genes, RAPD-genotyping

## Abstract

*Acinetobacter* spp. has gained fame from their ability to resist difficult conditions and their constant development of antimicrobial resistance. This study aimed to investigate the prevalence, susceptibility testing, OXA carbapenemase-encoding genes, and RAPD-genotyping of multidrug resistant *Acinetobacter baumannii* incriminated in hidden community-acquired infections in Egypt. The antimicrobial susceptibility testing was assessed phenotypically using Kirby–Bauer disk diffusion method. Also, Modified-Hodge test (MHT) was carried out to detect the carbapenemases production. Multiplex-PCR was used to detect the carbapenemase-encoding genes. Furthermore, the genetic relationship among the isolated strains was investigated using RAPD fingerprinting. The bacteriological examination revealed that, out of 200 Gram-negative non-fermentative isolates, 44 (22%) were identified phenotypically and biochemically as *Acinetobacter* spp. and 23 (11.5%) were molecularly confirmed as *A.* *baumannii*. The retrieved *A.* *baumannii* strains were isolated from urine (69%), sputum (22%), and cerebrospinal fluid (csf) (9%). The isolated *A. baumannii* strains exhibited multidrug resistance and the production rates of carbapenemases were 56.5, 60.9, and 78.3% with meropenem, imipenem, and ertapenem disks, respectively. The *bla*_OXA-24_-like genes were the most predominant among the tested strains (65.2%), followed by *bla*_OXA-23_ (30.4%) and *bla*_OXA-58_ (17.4%), in addition, the examined strains are harbored IMP, VIM, and NDM genes with prevalence of 60.9, 43.5, and 13%, respectively, while KPC and GES genes were not detected. RAPD-PCR revealed that the examined strains are clustered into 11 different genotypes at ≥90% similarity. Briefly, to the best of our knowledge, this study is the first report concerning community-associated *A. baumannii* infections in Egypt. The high prevalence of hidden multidrug-resistant (MDR) and extensively drug-resistant (XDR) *A.* *baumannii* strains associated with non-hospitalized patients raises an alarm for healthcare authorities to set strict standards to control the spread of such pathogens with high rates of morbidity and mortality.

## 1. Introduction

The bacterium *A. baumannii* is a Gram-negative, aerobic, coccobacilli that is marked to the greatest extent as being significant to healthcare-associated pneumonia, blood, cerebrospinal fluid (csf), and urinary tract infections with high mortality and transmission rates and resistance to multiple classes of antimicrobials [1,2,3]. Although it is still considered rare, community-acquired *A. baumannii* infections have been reported primarily in equatorial and sub-equatorial regions [4,5]. Recently, an increase in community-acquired *Acinetobacter* infections is being reported [6,7,8]. Health settings-acquired *A. baumannii* showed higher resistance to antibacterial drugs than community-acquired pathogens, despite these mortality rates still being very high (64%) [5,9]. Misidentification of *Acinetobacter* spp. is a well-documented and accurate genus or species identification, which possess a challenge to microbiologists even with commercially-available kits like API 20NE and Vitek2 systems [10,11]. Occasionally, *Burkholderia pseudomallei* is misidentified as *Acinetobacter* spp. and *A. baumannii* as *Alcaligenes faecalis* [12,13]. Furthermore, the *A. calcoaceticus–A. baumannii* complex (i.e., genomospecies 1, 2, 3, and 13) share the majority of biochemical properties and are hard to discriminate based on conventional or commercial identification schemes, but they differ in clinical features [14,15].

The efflux pump, many types of β-lactam degrading enzymes, and small membrane porins are involved in the natural resistance of *A. baumannii* to a wide range of antimicrobials [16,17]. *Acinetobacter* spp. is naturally competent with high natural transformation rates that have an essential role in antibiotic resistance genes gaining and distribution [18,19]. For a long time, carbapenem was the best drug to control *A. baumannii* infections; however, the evolved carbapenem resistance due to carbapenemase limited its efficacy [20]. In *A. baumannii,* three types of β-lactamases have been reported as belonging to class A, B, and D of Ambler classification: class A including beta-lactamases inhibited by clavulanic acid such as TEM and SHV, and clavulanic acid insensitive enzymes like KPC and GES; class B including Metallo β-lactamases such as VIM, IMP, and NDM; and class D including oxacillinases [21,22,23]. Ambler class D β-lactamases are serine hydrolases that have the ability to hydrolyze oxacillin and are called oxacillinases (OXA). According to their protein primary structure, oxacillinases are grouped into several families or subclasses like *bla_OXA-23_, bla_OXA-24_, bla_OXA-40_, bla_OXA-51_*, *bla_OXA-58_*, *bla_OXA-143_*, and *bla_OXA-23_* families [24]. The persistence of OXA-51 gene family is found in *A. baumannii* strains and believed to be intrinsic to this genomic species, but recently, this was detected in another species, perhaps due to horizontal gene transfer [25,26]. *A. baumannii* can easily develop resistance to a new lactam ring containing antibiotics due to the presence of this variety of OXA enzymes. Therefore, *A. baumannii* has become one of the problematic pathogens with no or very few options for eradication [27]. Given the importance of class D carbapenemase, much research was conducted addressing their genetic variation, enzymatic characterization, prevalence, and extent of antibiotic resistance throughout the globe [28,29].

Although *A. baumannii* is not a frequent cause of community-acquired infections, the capacity to rapidly develop resistance mechanisms to antibiotics and the fulminant clinical presentation (with a mortality rate around 50%) makes this pathogen an important health problem in the community setting [30]. This study is concerned with the prevalence of the hidden and/or unidentified *A. baumannii* among community-acquired infections caused by Gram-negative, non-fermenting bacterial pathogens in Egypt. We aim to analyze their antimicrobial susceptibility pattern, evaluate the incidence rates of different types of carbapenemases, and check genetic relatedness between the isolates.

## 2. Results

### 2.1. Identification and Prevalence of the Hidden Community-Acquired A. baumannii

In this study, 200 (9.5%) Gram-negative, non-fermentative strains were selected among 2108 Gram-negative isolates identified at mega laboratories over the Suez Canal and Nile Delta regions in Egypt (1044 urine culture, 108 pus, 537 sputum, 322 blood, 23 cerebrospinal fluid, and 74 wound) from August 2017 to October 2018. The 200 strains were preliminary inoculated on MacConkey agar, blood agar, Acinetobacter agar, and CHROM agar media. Out of the 200 strains, 44 (22%) showed *Acinetobacter* spp. differential growth on agar media. Among the 44 strains, 29 (66%) were identified as *A. baumannii* by API 20 NE bacterial identification system. The intrinsic OXA-51 of *A. baumannii* was found in only 23 of the 44 strains. For the 23 isolates, the 16S-23S region of ribosomal RNA sequence analysis showed that 23 of the 44 strains having 99.89 to 100% identity to *A. baumannii*.

Table 1 elucidates the dissemination of Gram-negative non-fermentative isolates according to sample source and gender. The Gram-negative non-fermentative isolates were most prevalent in urine (38.5%), sputum (20.5%), wound (20%), blood (11%), pus (6%), and cerebrospinal fluid (csf) (4%). The most prevalent Gram-negative non-fermentative among the 200 isolates were *Pseudomonas aeruginosa* 156 (78%) followed by *A. baumannii* 23 (11.5%), *A. lwoffii* 7 (3.5%), *A. calcoaceticus* 6 (3%), *Burkholderia cepacia* 3 (1.5%), *Stenotrophomonas maltophilia* 3 (1.5%), and *A. nosocomialis* 2 (1%). *P. aeuroginosa* was most prevalent in urine samples 57 (28.5%) followed by wound 35 (17.5%), sputum 31 (15.5%), blood 18 (9%), pus 10 (5%), and csf 5 (2.5%). The distribution of *P. aeuroginosa* was higher in male patients (47%) than in female patients (31%) (Table 1). 

As shown in Table 1, the 23 *A. baumannii* isolates were predominantly isolated from urine samples 16 (69.6%), sputum 5 (21.7%), and csf 2 (8.7%). Twenty-one (92.3%) *A. baumannii* isolates were collected from female patients: from urine 16 (76.2%), sputum 4 (19%), and csf 1 (4.8%). In male patients, 2 (8.7%) isolates were obtained, one isolate was collected from sputum and one from csf. The total age (of both male and female patients recorded an average of (±SD) 41.5 ± 16.23, 38.8 ± 28.40, and 28.5 ± 33.20 from urine, sputum, and csf sample origin (Table 2).

### 2.2. Antimicrobial Susceptibility Profiles of A. baumannii

**Figure 1:**
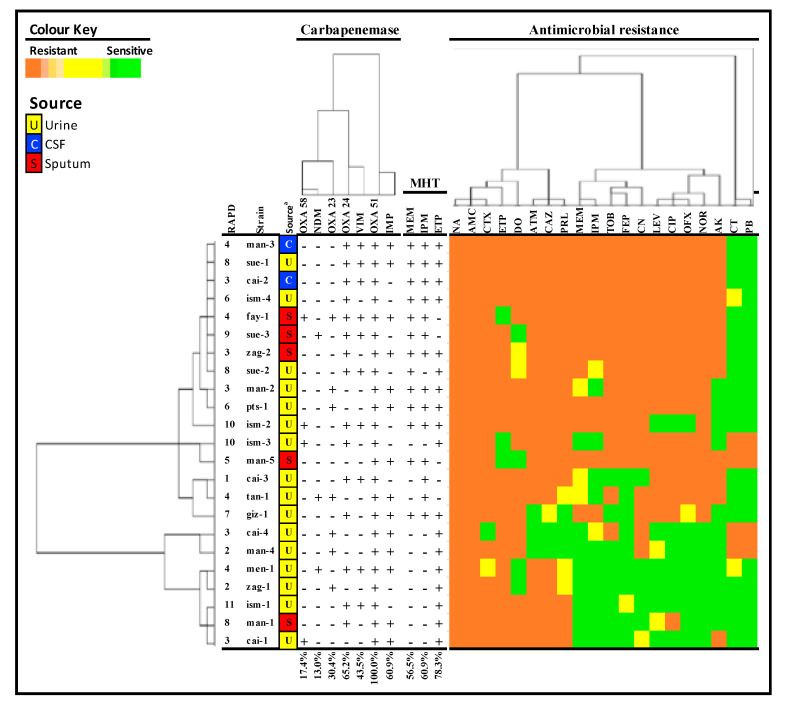
Random Amplification of Polymorphic DNA (RAPD) type, strain, source, carbapenemases, antimicrobial susceptibility, and MHT test of the 23 A. *baumannii* isolates. Individual isolates showing hierarchical clustering of isolates and factors. Binary factor (carbapenemase genes) indicated by presence (+) and absence (-); however, ordinal factor (antibiotics) indicating resistance by orange color, intermediate (yellow), and sensitive (green). Abbreviations: tobramycin (TOB), gentamycin (CN), amikacin (AK), norfloxacin (NOR), levofloxacin (LEV), ciprofloxacin (CIP), nalidixic acid (NA), ampicillin (AMP), piperacillin (PRL), amoxicillin/clavulanic acid (ATM), ceftazidime (CAZ), cefepime (FEP), cefotaxime (CTX), meropenem (MEM), imipenem (IPM), doripenem (DO), ertapenem (ETP), colistin (CT), and polymyxin B (PB).

Antimicrobial susceptibility profile was determined using 20 antibiotics, the susceptibility profiles were different in each isolate (Figure 1). The antimicrobial susceptibility profiles of *A. baumannii* were represented in Table 3, with the difference between S, R, and I assessed statistically with different antibiotics at 0.05 level. All the examined *A. baumannii* strains were resistant to a minimum of one antimicrobial agent in three or more antimicrobial categories (multidrug-resistant (MDR)), of which 19 (82.6%) were resistant to a minimum of one antimicrobial agent in all but two or less antimicrobial categories (extensively drug-resistant (XDR)) (Figure 1). As shown in Table 3, the strains, collectively, were resistant to oxacillin, nalidixic acid, and amoxicillin/clavulanic acid. The resistance rates to quinolones—ciprofloxacin, ofloxacin, levofloxacin, and norfloxacin were 69.6, 60.9, 65.2, and 69.6%, respectively. Resistance rates to aminoglycosides—tobramycin, gentamycin, and amikacin were 65.2, 73.9, and 52.2%, respectively. Cephalosporins resistance rates were 87, 91.3, and 56.5% for ceftazidime, cefotaxime, and cefepime, respectively. Carbapenem resistance showed 52.2, 47.8, 87, and 73.9% against meropenem, imipenem, ertapenem, and doripenem, respectively. The polypeptides polymyxin B and colistin were the most active antimicrobials agents against the studied *A. baumannii* with a sensitivity of 82.6% and 73.9%, respectively (Table 3).

### 2.3. Prevalence of Carbapenemase Producers

Modified Hodge Test (MHT) was carried out to detect the carbapenemases production in *A. baumannii* strains (*n* = 23), the results revealed that the production rates were 56.5, 60.9, and 78.3% with meropenem, imipenem, and ertapenem disks, respectively (Figure 1). Collectively, all the tested strains were MHT positive to a minimum of one carbapenem species used in the test.

### 2.4. Prevalence of Carbapenemase Encoding Genes

**Figure 2:**
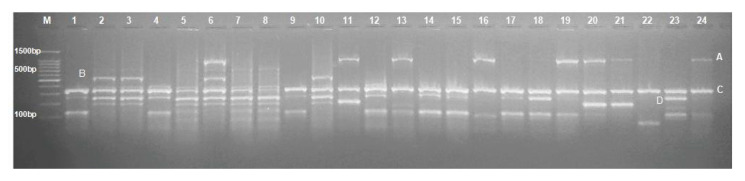
Multiplex PCR identification of OXA carbapenemase gene families in *A. baumannii* under investigation. A, B, C, and D mark for *bla*_OXA-23_ (1058 bp), *bla*_OXA-58_ (599 bp), *bla*_OXA-51_ (353 bp), and *bla*_OXA-24_ (246 bp) fragments, respectively. (M) indicates 100 bp Marker from Genetex, *A. baumannii* strains in the study from lane 1 to 23, and lane 24 represent *bla*_OXA-23_ positive control strain.

The multiplex-PCR revealed that *bla*_OXA-24_-like genes were the most predominant among the tested strains (65.2%), followed by *bla*_OXA-23_ (30.4%) and *bla*_OXA-58_ (17.4%), as described in Figure 1 and Figure 2. Furthermore, the prevalence of NDM, VIM, and IMP genes families within the tested strains was 13, 43.5, and 60.9%, respectively (Figure 1).

### 2.5. Random Amplification of Polymorphic DNA (RAPD) PCR Fingerprinting

**Figure 3:**
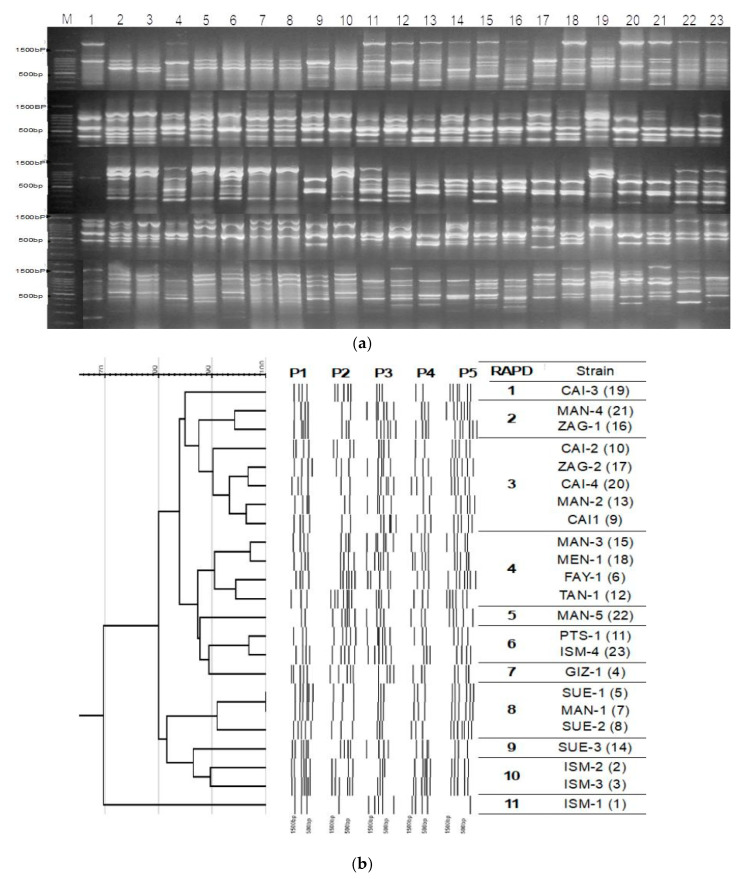
RAPD genotyping of the strains. (**a**) Combined RAPD patterns of 23 clinical *Acinetobacter* isolates obtained after electrophoresis of the PCRs with five different primers individually. For each primer, five RAPD gels were combined into one image. (**b**) RAPD dendrogram was constructed with GelJ cluster analysis software by Unweighted Pair Group Method with Arithmetic Mean UPGMA). Percentages of similarity and primers used as presented in Table 3 on the top line of the dendrogram. RAPD type and strain code as in Table 3 and strain numbers as presented in Figure 3a appear on the right (between parenthesis).

Ten RAPD primers (Table 4) were tested for all the 23 strains, five were chosen according to their reproducible and discriminating powers. The RAPD typing of the 23 community-acquired isolates has been performed by using all five RAPD primers (P1, P2, P3, P4, and P5) (Table 4). For each strain, five RAPD gels were combined into one single lane (Figure 3a). Each RAPD PCR generated up to 8 fragments per strain. The cumulative patterns yielded approximately 35 bands with fragments extended from 500 to 2000 bp per strain that were used for cluster analysis. Construction of the dendrogram was performed using GelJ cluster analysis software, the Dice coefficient and Unweighted Pair Group Method with Arithmetic Mean (UPGMA) method were used for analysis at ≥90 percentages similarity. In Figure 3b, RAPD discriminated 11 genetically distinct groups for the 23-community acquired *A. baumannii.* However, antibiotic resistance profiles were different between the isolates within the same group (Figure 1).

## 3. Discussion

The rise and proliferation of antibiotic-resistant pathogenic microorganisms are of incredible worry to clinicians. Since the original depiction of a penicillin-inactivating catalyst in *Escherichia coli*, the furious 75-year battle against these microbes has been alluded to as an “unwinnable war” [31]. Recent clinical consideration has concentrated on the expanding rate of lactose non-fermenting, Gram-negative pathogens liable for health settings-gained infections [31]. In this gathering, *Acinetobacter* species are rising as pathogens that now and again cause infections in patients in intensive care units [32]. In this kind of bacteria, protection from different classes of anti-infection agents genuinely bargains with the capacity to treat patients who are tainted with these pathogens. Hence, for the immunocompromised host, current efficient therapy is a matter of survival.

In the existing study, 44 (22%) isolates were preliminarily identified as *Acinetobacter* spp., using differential growth morphology and biochemical tests, from 200 Gram-negative, non-fermentative strains collected between October 2017 and August 2018 with several types of infections from different private and governmental laboratories through Egypt. Twenty-nine of the 44 (65.9%) strains were identified biochemically as *A. baumannii* by the API 20NE system. It is widely accepted that identification of non-fermenting, Gram-negative bacilli is usually carried out by commercial identification systems such as the API 20 NE [10,11].

*Acinetobacter* spp. characterization to the genospecies level using phenotypic techniques is difficult, especially identifying strains belonging to *A. baumannii* [33]. In this work, PCR detection of *A. baumannii* specific OXA-51 was used to confirm the identification of *A. baumannii*, 23 isolates showed the presence of *bla*OXA-51-like genes and was confirmed by 16s–23s rRNA region sequencing. Turton et al. (2006) provide evidence that *A. baumannii* can be identified by the detection of *bla*OXA-51 by PCR [26]. However, a plasmid-mediated *bla*OXA-51-like carbapenemase has been found in other genomic species rather than *A. baumannii,* and the accuracy of using *bla*OXA-51 detection as a tool for differentiating *A. baumannii* from other *Acinetobacter* species has been argued [34]. The sequencing of bacterial 16S-23S rRNA intragenic spacer region has proved useful in taxonomic studies to distinguish between different species of common genus [35].

During the present work, the most prevalent Gram-negative, non-fermentative isolates were *Pseudomonas aeruginosa* 156 (78%) followed by *A. baumannii* 23 (11.5%), *A. lwoffii* 7 (3.5%), *A. calcoaceticus* 6 (3%), *Burkholderia cepacia* 3 (1.5%), *Stenotrophomonas maltophilia* 3 (1.5%), and *A. nosocomialis* 2 (1%). Several studies showed that the highest prevalent Gram-negative non-fermentative pathogen in hospital-acquired infections is *P. aeruginosa* (50–88%) followed by *A. baumannii* (5–35%) [36,37,38].

In the current work, *A. baumannii* was isolated from urine (69.6%), followed by sputum (21.7%) and csf (8.7%). In studies on community-acquired *A. baumannii* in China, Taiwan, and Australia, 63.8% of cases were pneumonia and 36.2% cases were bacteremia [39]. Falagas et al. (2007) reviewed 26 case reports regarding 43 non-hospitalized patients having *A. baumannii* infections; from those, 88.4% pneumonia, 4.7% meningitis, 4.3% tissue infection, 4.3% ocular infection, and 4.3% endocarditis [39]. In our results, the highest percentage of isolation was from female patients (91.3%), followed by male patients (8.7%). Contrary to our results, Rebic et al. (2018) observed that *A. baumannii* infection was higher in male patients (58%) than female patients (42%) in a study that included 622 in- and out-patients in a Bosnian hospital [40]. These differences may be due to the randomized sampling pattern for our collected isolates without the concentration on specific symptoms or cases. Interestingly, Pires et al. (2020) found that female mice were more susceptible to *A. baumannii* pneumonia [41].

This study highlighted the importance of *Acinetobacter* spp. as an important community-acquired pathogen in contrast to the study done by Basuda K. and Medhabi S. (2013), which was carried out in a hospital setting and found that the predominant *Acinetobacter* spp. were *A.* *calcoaceticus* (42%) and *A. baumannii* (34%), and that both developed resistance to commonly-used antibiotics [14]. Recently, an exponential elevated rate of community-acquired *A. baumannii* cases has been reported that led to increased awareness of the danger of this type of infection with high pathogenicity and death rates, especially in equatorial and sub-equatorial geographical regions [39]. However, *Acinetobacter* spp. true incidence among Gram-negative, non-fermenting pathogens circulating the community has not been reported, to the best of our knowledge. Importantly, all of *Acinetobacter* spp. identified in this work were unidentified by the collecting lab, failure, and/or misidentification of *Acinetobacter* spp. with other gram-negative bacteria is common [12,13,15].

The small size and distribution of *A. baumannii* outer membrane porins are responsible for the decreased membrane permeability and may explain their effective, de facto higher resistance to certain antimicrobials [42]. Resistance to aminoglycosides like amikacin was 52.2% within the studied strains, which is close to other studies on *A. baumannii* from Egypt with 68.5% resistance [43]. *A. baumannii* cephalosporins resistance was similar to other studies in Egypt, Saudi Arabia, and India, where it ranges from 60 to 100% [44,45,46].

The resistance of the strains in this work to quinolones, nalidixic acid, and ciprofloxacin was close to studies from Algeria, Egypt, and Saudi Arabia [44,45,46]. *A. baumannii* strains’ resistance to carbapenems like meropenem and imipenem (65.2 and 56.5%, respectively) were comparable to other studies in Egypt (57.5 and 65%) and Saudi Arabia (50 and 48.1%) [44,45]. The incidence of carbapenem resistance is becoming a serious problem in *A. baumannii* worldwide. A plethora of reports on carbapenem resistant *A. baumannii* exists, which have been associated with carbapenemases production [47,48,49].

In this work, carbapenemases production, as measured by MHT, demonstrated that 56.9, 60.9, and 78.3% of the strains produce carbapenemase inhibiting either meropenem, imipenem, or ertapenem, respectively. Therefore, all *Acinetobacter* isolates were phenotypically positive carbapenem producers for at least one type of carbapenem disk in the MHT test. In another study from Egypt, 60% of *A. baumannii* were positive for carbapenemases production by MHT assay using meropenem disks [49]. Recently, the accuracy of MHT was urged, suggesting that carbapenem resistance of an isolate is more likely correlated to the type of carbapenem produced rather than the level of production [50,51,52].

The persistence of OXA-51 gene family are found in all tested *A. baumannii* strains and believed to be intrinsic to this genomic species, but recently this was detected in another species perhaps due to horizontal gene transfer [25,26]. *A. baumannii* can easily develop resistance to a new lactam ring containing antibiotics due to the presence of this variety of OXA enzymes. In the current study, among the 23 carbapenem-resistant strains, 30.4% strains were detected as positive for the presence of *bla*OXA-23-like genes, 65.2% for *bla*OXA-24-like genes, and 17.4% for *bla*OXA-58-like genes. This differed from the previously reported prevalence of OXA type carbapenemases in 40 carbapenem-insensitive *A. baumannii* isolates from hospitalized patients collected from Egypt in 2014, which were 50, 7.5, and 5%, respectively [45]. This difference in OXA carbapenemases prevalence may indicate different genetic lineage between community-acquired and hospital-acquired *A. baumannii* in Egypt [45]. In Taiwan, the prevalence of *bla*OXA-23-, *bla*OXA24-, and *bla*OXA-58-like genes was 28.69, 90.91, and 0%, respectively [53]. The prevalence of other types of carbapenemases, such as *bla*NDM, *bla*VIM, *bla*IMP was similar to other studies in Egypt, where it was found that the incidence of *bla*NDM was 39.3%, *bla*IMP 95.7%, and *bla*VIM 93% [54,55,56].

All profiles recognized in this investigation were ordered into 11 groups that were seen in 23 strains by utilizing RAPD-PCR demonstrating high genotypic varieties. Various examinations have been coordinated on genotyping *A. baumannii* strain by different genotyping techniques, and various genetic profiles have been represented in various regions. The findings of this study are similar to that of studies reported by Asadian et al. (2019), who were also able to cluster *A. baumannii* strains based on their genetic relatedness using RAPD-PCR [57]. The assorted variety of genetic patterns among the isolates is probably due to a wide assortment of sampling locations, otherwise several strains with identical genetic pattern and common origin would have been observed. It is also noticeable that strains originated from different infection sites were grouped genetically in the same cluster, suggesting the same origin and increase of the danger of multiple infection capabilities for such genotypes. Distinguishing proof of a satisfactory degree of decent genetic variety among the strains by this strategy utilizing five consolidated RAPD oligos demonstrates that this technique is valuable for examining and typing *A. baumannii,* and strains with various roots can be arranged into various clusters. This agreed with Koeleman et al. (1998), who proposed using at least 5 RAPD primers to better discriminate genetic diversity between bacterial isolates [58].

## 4. Materials and Methods

### 4.1. Ethical Approval

The research was ethically approved by the Faculty of Science—Suez Canal University (2017 MS518) and is in compliance with the Helsinki Declaration of 1964 and the resulting revisions or comparative moral guidelines. This research was waived from written informed consent/assent/parental permission, and the patients were informed orally.

### 4.2. Study Design and Bacterial Isolates

A sum of 200 non-repetitive, Gram-negative, non-fermentative isolates was selected from 2108 isolates from non-hospitalized (for the last 12 months) symptomatic patients from private medical and microbiological analysis laboratories across Egypt during a period of 11 months from September 2017 to August 2018. These bacteria were originally isolated from sputum, urine, blood, cerebrospinal fluid. Isolates (*n* = 200) were streaked on MacConkey agar (OXOID, Basingstoke, England), Leed Acinetobacter agar (Hardy Diagnostics, Santa Maria, CA, USA), and CHROMagar (Oxoid, Basingstoke, England), and incubated under aerobic conditions at 37 °C for 48 hours. Gram staining, motility, oxidase, peroxidase, and oxidative-fermentation reactions were performed according to standard techniques [59]. API 20NE bacterial identification system (Biomerieux, Marcy-l’Étoile, France) was used for species identification.

### 4.3. Molecular Identification of A. baumannii

*A. baumannii* strains, identified by API 20NE system, were confirmed by PCR detection of *bla*OXA-51-like genes, according to *Evans* et al. (2008) [60] and ribosomal RNA 16S–23S sequencing, based on the method of Chang et al. (2005) [33].

### 4.4. Antimicrobial Susceptibility Testing of A. baumannii

The sensitivity of *Acinetobacter* isolates to different families of antibiotics were inclusive of: aminoglycosides—tobramycin (10 µg), amikacin (30 µg), and gentamicin (10 µg); beta-lactam combinations—piperacillin/tazobactam (100/10 µg) and amoxicillin/clavulanic acid (20/10 µg); quinolones—norfloxacin (10 µg), levofloxacin (5 µg), Ofloxacin (5 µg), nalidixic acid (30 µg), and ciprofloxacin (5 µg); monobactams—aztreonam (30 µg); cephalosporins—cefepime (30 µg), ceftazidime (30 µg), cefotaxime (30 µg); carbapenems—doripenem (10 µg), ertapenem (10 µg), imipenem (10 µg), meropenem (10 µg), and Polypeptides—polymyxin B (300 U) and colistin (10 µg) were examined by Kirby–Bauer disk diffusion approach [61]. *Escherichia coli* ATCC 25922 was used in quality control. Antibiotic discs were obtained from Oxoid, UK. Colistin and polymyxin resistance was confirmed by MIC assay [61].

### 4.5. Screening of Carbapenemases Production

Screening carbapenemases production was performed using ertapenem, imipenem, and meropenem (10 μg) discs by the Modified Hodge Test (MHT) in consonance with Shivaprasad et al. (2014) [62]. *Acinetobacter baumannii* NCTC 13301 and *Escherichia coli* ATCC 8739 were used as positive and negative controls, respectively.

### 4.6. Molecular Detection of Carbapenemase Encoding Genes

Purified bacterial colonies were resuspended in sterile saline (200 μL). Genomic DNA was obtained by the bacterial-genomic DNA extraction kit (BioVision, New Minas, NS, Canada) following manufacturer directions. PCR was used to detect *bla*OXA-51 gene in the isolates as described previously [60]. Multiplex PCR was used for the detection of *bla*OXA-23, *bla*OXA-24, *bla*OXA-51, and *bla*OXA-58 genes as previously described with slight modification [63]. Different *bla*OXA-23 primers used in the multiplex PCR for better discrimination [64]. PCR visualization of *bla*IMP, *bla*VIM, *bla*NDM, *bla*GES, and *bla*KPC was performed as described previously [63,65]. Primers and fragments’ lengths are presented in Table 3. *bla*OXA-58 and *bla*OXA-24 amplicons were confirmed by direct PCR sequencing.

### 4.7. RAPD—Genotyping of A. baumannii

Ten decanucleotide primers obtained from RAPD kit (Eurofins Biodiagnostics) and used in this study (Table 3). RAPD analysis was accomplished in accordance with Zhang et al. (2008) [66]. For cluster analysis, a DNA fingerprint java application analysis software (GelJ) was used (downloaded from https://sourceforge.net/projects/gelj/) [67]. UPGMA method was applied in cluster algorism and dendrogram creation using Dice as a similarity index. A similarity index of ≥90% was used for clustering genetically-related isolates.

### 4.8. Statistical Analysis

Data were checked for normality using Shapiro–Wilk normality testing at *p* < 0.05. Parametric data were presented as mean followed by standard deviation. Non-parametric data were presented as number and percentages. Differences in parametric data were checked using one-way analysis of variance (ANOVA) at *p* < 0.05. Non-parametric data were checked using Chi squared, and Kruskal–Wallis test statistic to differentiate between (sensitive, intermediate, resistant). Dendrogram were plotted by hierarchical clustering analysis of isolates and factors, both binary factor (carbapenemase genes) and ordinal factor (antibiotics) using Ward’s method and squared Euclidean distances. Statistical analyses were carried by IBM SPSS version 23.0 for Mac OS. (Table 4).

## 5. Conclusions

In this work, we identified multidrug-resistant strains of *A. baumannii* from community-acquired infections in Egypt. The isolated *A. baumannii* strains exhibited multidrug resistance with the production of carbapenemases. The blaOXA-24-like genes were the most predominant among the tested strains, followed by blaOXA-23 and blaOXA-58. In addition, the examined strains harbored IMP, VIM, and NDM genes. Genetic relatedness studies revealed 11 different genotypes with more than 90% similarity. The current study provided phenotypic and genotypic characterization data of community-acquired *A. baumanii*. Further studies are needed to assess other resistance genes, the potential virulence factors of these community-acquired strains, and the severity of the clinical outcome associated with these strains.

The findings of this research essentially encourage continual precise monitoring and reporting of the incidences and antimicrobial susceptibility of community-associated *Acinetobacter* spp. to help clinicians in the containment of these life-threatening infections.

## Figures and Tables

**Table 1:** Distribution of Gram-negative non-fermentative isolates according to sample source and patient gender (*n* = 200).

	Sample Origin	Gender	Urine	Sputum	Pus	Blood	Wound	CSF *	Total	Collecting Lab. Identification %
Strain									n (%)	
*Pseudomonas aeruginosa*		Male Female Total	36	18	6	10	21	3	94 (47)	100%
			21	13	4	8	14	2	62 (31)	
			57	31	10	18	35	5	156 (78)	
*Acinetobacter baumannii*		Male Female Total	0	1	-	-	-	1	2 (1)	unidentified
			16	4	-	-	-	1	21 (10.5)	
			16	5	-	-	-	2	23 (11.5)	
*Acinetobacter lwoffii*		Male Female Total	2	3	-	1	-	-	6 (3)	unidentified
			1	0	-	0	-	-	1 (0.5)	
			3	3	-	1	-	-	7 (3.5)	
*Acinetobacter nosocomialis*		Male Female Total	-	-	1	1	-	-	2 (1)	unidentified
			-	-	0	0	-	-	0	
			-	-	1	1	-	-	2 (1)	
*Acinetobacter calcoaceticus*		Male Female Total	0	1	1	-	2	-	4 (2)	unidentified
			1	0	0	-	1	-	2 (1)	
			1	1	1	-	3	-	6 (3)	
*Burkholderia cepacia*		Male Female Total	-	0	-	-	1	-	1 (0.5)	unidentified
			-	1	-	-	1	-	2 (1)	
			-	1	-	-	2	-	3 (1.5)	
*Stenotrophomonas maltophilia*		Male Female Total	-	-	-	0	-	1	1 (0.5)	unidentified
			-	-	-	2	-	0	2 (1)	
			-	-	-	2	-	1	3 (1.5)	
Total		Male Female Total	38	23	8	12	24	5	110 (55)	
			39	18	4	10	16	3	90 (45)	
			77	41	12	22	40	8	200	

* cerebrospinal fluid.

**Table 2:** Distribution of *A. baumannii* isolates according to sample source.

Variables		Sample Origin								Sign
		Urine		Sputum		CSF		Total		
		n	%	n	%	n	%	n		
Gender	Male	0	0.0	1	50	1	50	2		0.009 *
	Female	16	76.2	4	19.0	1	4.8	21		
	Total	16	69.6	5	21.7	2	8.7	23		
Age	Male (mean ± SD)	0.0		18.0 ± 19.02		52.0 ± 19.21		35.0 ± 13.58		0.049 *
	Female (mean ± SD)	41.5 ± 4.80		44.0 ± 9.60		5.0 ± 19.21		30.17 ± 7.34		
	Total (mean ± SD)	41.5 ± 16.23		38.8 ± 28.40		28.5 ± 33.20		39.8 ± 19.90		0.695 ns
Age groups	0–15 years	0	0.0	1	50.0	1	50.0	2		0.333 ns
	16–30 years	7	87.5	1	12.5	0	0.0	8		
	31–55 years	4	66.7	1	16.7	1	16.7	6		
	>56 years	5	71.4	2	28.6	0	0.0	7		
	Total	16	69.6	5	21.7	2	8.7	23		

* significant at *p* < 0.05; ns, non-significant at *p* > 0.05.

**Table 3:** Antimicrobial resistance profiles of *A. baumannii*.

Antimicrobial	Resistance (*n*) %			Sign.
	S	R	I	
Tobramycin	(8) 34.8%	(15) 65.2%	0	0.144 ns
Gentamycin	(5) 21.7%	(17) 73.9%	(1) 4.3%	<0.001 *
Amikacin	(11) 47.8%	(12) 52.2%	0	0.835 ns
Aztreonam	(3) 13%	(20) 87%	0	<0.001 *
Norfloxacin	(7) 30.4%	(16) 69.6%	0	0.061 ns
Levofloxacin	(6) 26.1%	(15) 65.2%	(2) 8.7%	0.003 *
Ofloxacin	(8) 34.8%	(14) 60.9%	(1) 4.3%	0.0004 *
Ciprofloxacin	(7) 30.4%	(16) 69.6%	0	0.061 ns
Nalidixic acid	0	(23) 100%	0	<0.001 *
Piperacillin/tazobactam	(3) 13%	(19) 82.6%	(3) 13%	<0.001 *
Oxacillin	0	(23) 100%	0	<0.001 *
Amoxicillin/clavulanic acid	0	(23) 100%	0	<0.001 *
Ceftazidime	(2) 8.7%	(20) 87%	(1) 4.3%	<0.001 *
Cefotaxime	(1) 4.3%	(21) 91.3%	(1) 4.3%	<0.001 *
Cefepime	(9) 39.1%	(13) 56.5%	(1) 4.3%	0.008 *
Meropenem	(8) 34.8%	(12) 52.2%	(3) 13%	0.070 ns
Imipenem	(10) 39.1%	(11) 47.8%	(2) 8.7%	<0.001 *
Ertapenem	(3) 13%	(20) 87%	0	<0.001 *
Doripenem	(5) 21.7%	(17) 73.9%	(1) 4.3%	<0.001 *
Colistin	(17) 73.9%	(4) 17.4%	(2) 8.7%	<0.001 *
Polymyxin B	(19) 82.6%	(4) 17.4%	0	0.002 *

* significant at *p* < 0.05; ns, non-significant at *p* > 0.05.

**Table 4:** Sequence, amplicon size and annealing temperature of oligos used in the study.

Oligo	Sequence (5′→3′)	Amplicon Size (bp)	Annealing Tmp (°C)	Reference
(16-23S ITS) P-1512F	GTCGTAACAAGGTAGCCGTA	607	60	[33]
(16-23S ITS) P-6R	GGGTTC/TCCCCA/GTTCRGAAAT			
*bla*OXA-69F (F)	CTAATAATTGATCTACTCAAG	975	48	[60]
*bla*OXA-69R (R)	CCAGTGGATGGATGGATAGATTATC			
*bla*OXA-51F (F)	TAATGCTTTGATCGGCCTTG	353	52	[63]
*bla*OXA-51R (R)	TGGATTGCACTTCATCTTGG			
*bla*OXA-23F (F)	GATGTGTCATAGTATTCGTCGT	1058		[64]
*bla*OXA-23R (R)	TCACAACAACTAAAAGCACTGT			
*bla*OXA-24F (F)	GGTTAGTTGGCCCCCTAAAA	246		[63]
*bla*OXA-24R (R)	AGTTGAGCGAAAAGGGGATT			
*bla*OXA-58F (F)	AAGTATTGGGGCTTGTGCTG	599		[63]
*bla*OXA-58R (R)	CCCCTCTGCGCTCTACATAC			
(*bla*IMP) IMP-F (*bla*IMP) IMP-R	GGAATAGAGTGGCTTAAYTCTC GGTTTAAYAAAACAACCACC	232	52	[63]
(*bla*VIM) VIM-F (*bla*VIM) VIM-R	GATGGTGTTTGGTCGCATA CGAATGCGCAGCACCAG	390		
(*bla*NDM) NDM-F (*bla*NDM) NDM-R	GGTTTGGCGATCTGGTTTTC CGGAATGGCTCATCACGATC	621		
(*bla*KPC) KPC-F (*bla*KPC) KPC-R	CGTCTAGTTCTGCTGTCTTG CTTGTCATCCTTGTTAGGCG	798		
(*bl*aGES) GES-F (*bla*GES) GES-R	ATGCGCTTCATTCACGCAC CTATTTGTCCGTGCTCAGGA	863	55	[65]
OPM-01 (P1)	GTTGGTGGCT	Variable	40	Eurofins Kit
OPM-07 (P2)	CCGTGACTCA			
OPM-09 (P3)	GTCTTGCGGA			
OPQ-06 (P4)	GAGCGCCTTG			
OPQ-08 (P5)	CTCCAGCGGA			
OPM-03 (P6)	GGGGGATGAG			
OPQ-02 (P7)	TCTGTCGGTC			
OPM-05 (P8)	GGGAACGTGT			
OPQ-04 (P9)	AGTGCGCTGA			
OPQ-10 (P10)	TGTGCCCGAA			
